# Outbreak investigation of echovirus 11 among neonates in two confinement centers, Petaling District, Malaysia, 2023

**DOI:** 10.1016/j.ijregi.2026.101023

**Published:** 2026-09-26

**Authors:** Jun Fai Yap, Soo Cheng Lee, Zaharah Zainuddin

**Affiliations:** 1 Molecular Pathology Unit, Cancer Research Centre, Institute for Medical Research, National Institutes of Health, Ministry of Health Malaysia, Shah Alam, Selangor, Malaysia; 2 Petaling District Health Office, Selangor State Health Department, Ministry of Health Malaysia, Shah Alam, Selangor, Malaysia; 3 Epidemiology Division, National Public Health Laboratory, Ministry of Health Malaysia, Sungai Buloh, Selangor, Malaysia

**Keywords:** Enterovirus, Epidemiology, Infants, Meningoencephalitis, Public health

## Abstract

**Objectives:**

In November to December 2023, an outbreak of echovirus 11 (E11) infection involving neonates was identified in two confinement centers in Malaysia. This public health investigation aimed to describe its control measures.

**Methods:**

An epidemiologic, environmental, and laboratory investigation was conducted among neonates and staff at the two confinement centers. Environmental assessments focused on hand hygiene, cleaning, cot spacing, feeding, bathing, and diaper handling. Clinical specimens were assessed using molecular tests.

**Results:**

Two concurrent E11 outbreaks occurred without an established epidemiologic link. Six cases occurred in center A and four in center B, with neonatal attack rates of 66.7% and 50.0%, respectively. Five neonates were managed for presumed sepsis, including two asymptomatic neonates. Three had features of meningoencephalitis; one presented with hand, foot, and mouth disease; and one died of septic shock with severe pneumonia. Environmental assessment identified transmission risks, including close cot spacing, consecutive handling of neonates, shared bathing equipment, and inadequate hand hygiene or environmental cleaning.

**Conclusion:**

Similar infection prevention and control gaps in both confinement centers might have facilitated viral transmission, highlighting the need for strengthened hygiene practices and staff training. Asymptomatic and severe E11 infections emphasize the importance of early detection and appropriate laboratory investigations.

## Highlights

• A total of 10 neonates were laboratory-confirmed to have an echovirus 11 infection.
• Three neonates developed meningoencephalitis associated with echovirus 11.
• One neonate died from septicemic shock with severe pneumonia.
• Both confinement centers had similar infection prevention and control gaps.
• Poor hand hygiene and environmental factors posed viral transmission risks.

## Introduction

Confinement centers are private establishments, typically repurposed from residential or commercial properties, that provide continuous care to neonates and their postpartum mothers for up to 1 month after delivery. In Malaysia, reports indicate that staff at confinement centers often lack health care training, including proper infection prevention and control (IPC) measures [1,2]. Although some confinement centers employ trained nurses or midwives, many rely on untrained non–health care professionals who are familiar with traditional postpartum practices to provide maternal and newborn care [3].

Echovirus 11 (E11), part of the Enterovirus genus in the Picornaviridae family, has an incubation period of typically 4-7 days [4]. A wide variety of symptoms can be observed among infected individuals, with clinical manifestations depending on host factors such as age, sex, or immune status, as well as the specific subgroup of the viral strain involved. These non-specific symptoms are often self-limiting and include undifferentiated fever, diarrhea, exanthem, headache, cough, pleurodynia, irritability, lethargy, or acute flaccid paralysis. Life-threatening complications may occur, namely hand, foot, and mouth disease with fulminant hepatitis, sepsis, aseptic meningitis, encephalitis, myopericarditis, acute liver failure, or even lethal multi-system organ failure [5,6]. Although uncommon, fatal cases secondary to new variants of E11 have been reported recently in China, Japan, France, Italy, and the United Kingdom and Northern Ireland [[7], [8], [9], [10], [11], [12], [13]].

Despite the potential severity of echoviral infections, a relatively high proportion of patients exhibit no clinically apparent symptoms, displaying asymptomatic carriage with possible continual shedding of infectious E11 [14]. Although echoviral infections can affect individuals of all age groups, neonates in particular experience infection rates several times higher than those observed in older children and adults [15]. The underlying explanation is likely attributable to the increased expression of neonatal Fc receptors on intestinal enterocytes (the primary site of infection) and on microvascular endothelial cells lining the blood–brain barrier (a secondary site of infection) [16].

In Malaysia, E11 was the most common echovirus serotype circulating from 2003 to 2013, as determined by sequencing and phylogenetic analysis [17]. After alerts of E11 infections among neonates in European Union Member States throughout 2023, the World Health Organization recommended that countries actively investigate reported cases [18]. On November 27, 2023, the Petaling District Health Office (DHO) received notification of a laboratory-confirmed enterovirus case for contact tracing purposes in a confinement center. Subsequent active case-finding efforts revealed the extent of the epidemic, presenting as a confirmed E11 outbreak at the district level, involving several neonates across two confinement centers. Herein, we present a comprehensive epidemiologic investigation of the E11 outbreak in the Petaling District.

## Methods

### Epidemiologic investigation

This was a descriptive epidemiologic study of an outbreak involving two confinement centers (designated as confinement centers A and B) in the urban Petaling District, Selangor, Malaysia, between November 27 and mid-December 2023. The case definition included any neonate or staff member residing or working at either confinement center during the outbreak period, who developed symptoms suggestive of sepsis and was subsequently laboratory-confirmed positive for E11. Active case finding was conducted through interviews with staff members, parents of neonates, and visitors to the confinement centers to identify individuals with relevant symptoms and potential exposure. Passive case finding in the wider community was not conducted because all identified close contacts were confined within the two centers and no epidemiologically relevant contact with individuals from outside the confinement centers was identified. Sociodemographic characteristics, dates of symptom onset, and birth histories of the neonates were obtained through a review of confinement center records.

### Environmental investigation

An environmental investigation, including a walk-through survey, was conducted by a team of public health officers at both confinement centers on November 28, 2023. The assessment focused on environmental hygiene, facility layout, IPC practices, and potential areas of transmission. Particular attention was given to high-touch surfaces, communal areas, diaper-changing stations, handwashing facilities, and waste disposal areas. The adequacy and functionality of these facilities, as well as the movement patterns of staff, babies, parents, and visitors, were assessed. Cleaning and disinfection practices were reviewed, including the frequency of cleaning and adherence to national IPC guidelines. Caregiving practices were also observed, with an emphasis on hand hygiene, diaper handling, feeding practices, and disposal of potentially contaminated materials.

Interviews were conducted with key personnel from both confinement centers to obtain information on routine operations, outbreak response measures, visitor access policies, and staff illness history. Relevant documentation, cleaning schedules, visitor logs, and staff training records were also reviewed. Environmental risks identified during the assessment were documented and mapped to assess potential transmission pathways.

### Laboratory investigation

Rectal swabs were taken from all neonates at both confinement centers for enterovirus testing because enteroviruses are commonly shed through the gastrointestinal tract. This formed part of active case detection to facilitate the early identification and isolation of infected neonates and ensure timely medical intervention. Nasopharyngeal swab, cerebrospinal fluid (CSF) or serum were collected based on clinical indications and the attending paediatrician’s assessment. Serum specimens were collected from neonates with suspected severe E11 infection at the request of the attending paediatrician, while CSF specimens were collected from neonates presenting with neurological manifestations, as clinically indicated. Hand and rectal swabs were also collected from all staff members. These specimens were collected to identify possible transmission risks.

Clinical specimens were not collected from mothers because all mothers were asymptomatic and had no clinical features suggestive of E11 infection. Furthermore, mothers were restricted to their respective rooms and were not permitted to handle other neonates or move freely within the confinement centers. These restrictions limited their opportunities for direct contact with other neonates.

In accordance with established laboratory referral arrangements, specimens from hospitalised cases were referred to the Institute for Medical Research (IMR), whereas specimens collected from individuals in the community were referred to the National Public Health Laboratory (NPHL). At the time of specimen collection, three neonates had already been admitted to the hospital. Their clinical specimens were collected and tested at the IMR using real-time reverse transcription–polymerase chain reaction (RT-PCR) for detection of E11. The Petaling DHO collected specimens from asymptomatic neonates who were still present at the confinement centers at that time. These specimens were transported to the NPHL within 24 hours of collection for real-time RT-PCR testing. Specimen collection, handling, transportation, and laboratory testing were conducted in accordance with the respective laboratory protocols and the Guidelines for the Safe Transport of Clinical Specimens and Infectious Substances in Malaysia 2023.

At the NPHL, a multiplex real-time RT-PCR was performed for the initial detection of enteroviruses, targeting Enterovirus A71, Coxsackievirus A16, Coxsackievirus A6, and pan-enterovirus [19]. Specimens positive for pan-enterovirus were subsequently subjected to viral protein 1 (VP1) gene sequencing for the identification of E11 [20]. At the IMR, an in-house–developed singleplex real-time RT-PCR assay targeting E11 was performed for the detection of E11.

## Results

### Epidemiologic findings

A total of 10 neonates tested positive for E11. In confinement center A, six of the 13 occupants at risk (nine neonates and four staff members) were identified as cases (Table 1), resulting in an overall attack rate of 46.2%. All six cases were neonates, giving a neonatal attack rate of 66.7%. None of confinement center A’s staff members tested positive for E11. The cases had a mean age of 15 days at disease onset, ranging from 6 to 30 days.

**Table 1:** Characteristics of neonates who tested positive for E11 in two confinement centers (n = 10), Petaling District, Selangor, Malaysia, November to December 2023.

Case no.	Center	Sex	Gestational age	Duration of stay at confinement center before onset (days)	Day of life at onset or laboratory-confirmed (days)	Clinical presentation	Positive clinical specimens	Initial diagnosis	Status
1	A	Male	Term	18	20	Symptomatic	Rectal swab	Enteroviral infection (presumed meningitis)	Alive
							Cerebrospinal fluid		
2^a^	A	Male	Term	4	6	Symptomatic	Rectal swab	Enteroviral meningoencephalitis	Alive
							Cerebrospinal fluid		
3	A	Male	Term	12	14	Symptomatic	Rectal swab	Hand, foot, and mouth disease	Alive
4	A	Male	Term	10	10	Symptomatic	Rectal swab	Enteroviral meningoencephalitis	Alive
5	A	Male	Term	29	20	Symptomatic	Rectal swab	Presumed sepsis	Alive
6	A	Male	Term	30	30	Asymptomatic	Rectal swab	Presumed sepsis	Alive
7	B	Male	Term	6	7	Symptomatic	Serum	Severe septicemia secondary to pneumonia (multiorgan failure)	Deceased
8	B	Female	Term	28	15 days	Asymptomatic	Rectal swab	Presumed sepsis	Alive
9^a^	B	Female	Preterm (36 weeks 4 days)	19	25	Symptomatic	Rectal swab	Presumed sepsis	Alive
10^a^	B	Male	Term	31	33	Symptomatic	Rectal swab	Presumed sepsis	Alive

Abbreviation: E11, echovirus 11.

a Clinical specimens were sent to the Institute for Medical Research, whereas specimens from cases without ^a^ were sent to the National Public Health Laboratory.

In confinement center B, four of the 12 occupants at risk (eight neonates and four staff members) were identified as cases, resulting in an overall attack rate of 33.3%. All four cases were neonates, giving a neonatal attack rate of 50.0%. None of confinement center B’s staff members tested positive for E11. The cases had a mean age of 20 days at disease onset, ranging from 7 to 33 days. Although cases occurred concurrently in both confinement centers, no epidemiologic link was established between the six cases in confinement center A and the four cases in confinement center B.

All 10 infected neonates were of Chinese ethnicity, and the majority were male (80%). Two neonates were born to mothers with gestational diabetes mellitus, and most (60%) were delivered via lower segment cesarean delivery. Only one neonate was born preterm, at 36 weeks and 4 days of gestation, whereas the remaining nine were born at term. None of the affected neonates were from twin pregnancies.

Most (80%) cases were symptomatic, whereas two of 10 were asymptomatic. Nevertheless, all cases were hospitalized regardless of their symptom status. Half of the neonates were managed as presumed sepsis, including the two asymptomatic neonates; three had features of meningoencephalitis, and one presented with hand, foot, and mouth disease. Bacterial co-infection data were not collected during the public health investigation because assessment of bacterial co-infections was undertaken as part of the clinical diagnostic evaluation at the hospital, whereas the public health investigation focused primarily on the prevention and control of the E11 outbreak.

Two (20%) cases developed transaminitis leading to jaundice. CSF specimens were collected from hospitalized neonates with features of meningoencephalitis, and two cases tested positive for E11. All cases, except the fatal case, were discharged home without neurologic sequelae.

### Environmental findings

Both confinement centers were found to possess valid operating business licenses issued by the respective local councils. Alcohol-based handrub stations were available at both facilities, and the products were within their expiry dates at the time of inspection. However, neither center had a documented protocol for hand hygiene between handling individual babies.

IPC guidelines generally recommend maintaining more than 1m of spacing between patient beds to reduce the risk of droplet and contact transmission [21]. However, in confinement center A, six baby cots were placed in a single well-ventilated room, with approximately 30 cm spacing between adjacent cots. An adjacent room of similar size was also available and could accommodate up to four cots, allowing slightly greater spacing between them. In confinement center B, four baby cots were arranged side by side, with a similar spacing of approximately 30 cm.

Further observations in both confinement centers revealed several practices that could potentially increase the risk of communicable disease transmission. Neonates were bottle-fed consecutively by staff members, with no change of apron observed between babies. Neonates were also bathed consecutively in a single shared basin, without immediate disinfection of the basin between uses. Cleaning was performed using a commercially available antiseptic liquid, which was applied only at the end of each shift. Although disposable diapers were used for all neonates, soiled diapers were not disposed of immediately after each nappy change. No fever, diarrhea, or medical leave among staff members were reported during the same period.

### Laboratory findings

In confinement center A, 13 rectal swabs were collected, of which six tested positive for E11. In confinement center B, 12 rectal swabs were collected, of which three tested positive for E11. One serum specimen was collected from the neonate who subsequently died of septic shock with severe pneumonia, and the specimen tested positive for E11. Eight hand swabs were collected from staff members across both confinement centers, and all tested negative for E11.

### Prevention and control measures

Immediate control measures were implemented under the Prevention and Control of Infectious Disease Act 1988 (Act 342) to prevent further transmission. Confinement center B was temporarily closed on December 14, 2023, and confinement center A was closed on December 15, 2023, for a minimum period of 2 weeks, corresponding to twice the maximum incubation period of E11. No new neonates were received at either center during this period.

After the closure of both confinement centers, a comprehensive debriefing session was held with the management staff of both centers to review the outbreak response, discuss the challenges encountered during the investigation, and identify measures to strengthen IPC practices. The initial environmental walk-through identified several gaps, including the need for improved staff training on hand hygiene, environmental cleaning, and appropriate spacing between baby cots. The importance of establishing clear and standardized IPC procedures was, therefore, emphasized.

During the closure period, the Petaling DHO conducted follow-up visits to both centers to reinforce targeted interventions. These included structured staff education on hand hygiene, with an emphasis on the use of alcohol-based handrub between handling individual neonates; revised cleaning protocols; guidance on appropriate disinfectants; and advice on the immediate disposal of used diapers. The spatial arrangement of baby cots was also reviewed, with a recommended spacing of 1-2 m to support IPC practices.

In addition, a town hall session was conducted with operators of other confinement centers in Petaling District and representatives from the health authority. The session focused on sharing lessons learned from the outbreak and reinforcing health promotion practices to reduce the risk of similar outbreaks in other confinement centers. Findings from the IPC control measures were communicated to the Selangor State Health Department through situation reports. After the implementation of the interventions, both confinement centers underwent reassessment visits. Improvements were observed in hygiene practices, environmental cleanliness, and staff adherence to IPC measures.

## Discussion

This investigation identified 10 laboratory-confirmed E11 infections among neonates in two confinement centers in Petaling District, with cases occurring during the same period (Fig. 1). The clustering of cases over timein each center suggested that transmission had occurred within the respective confinement centers. Although no epidemiologic link was established between the two centers, both centers had similar potential risk factors for transmission, including close spacing between baby cots, consecutive handling of neonates by staff, shared bathing equipment, and gaps in hand hygiene and environmental cleaning practices. These similarities suggest that the conditions within both centers may have provided opportunities for E11 transmission.

**Figure 1:**
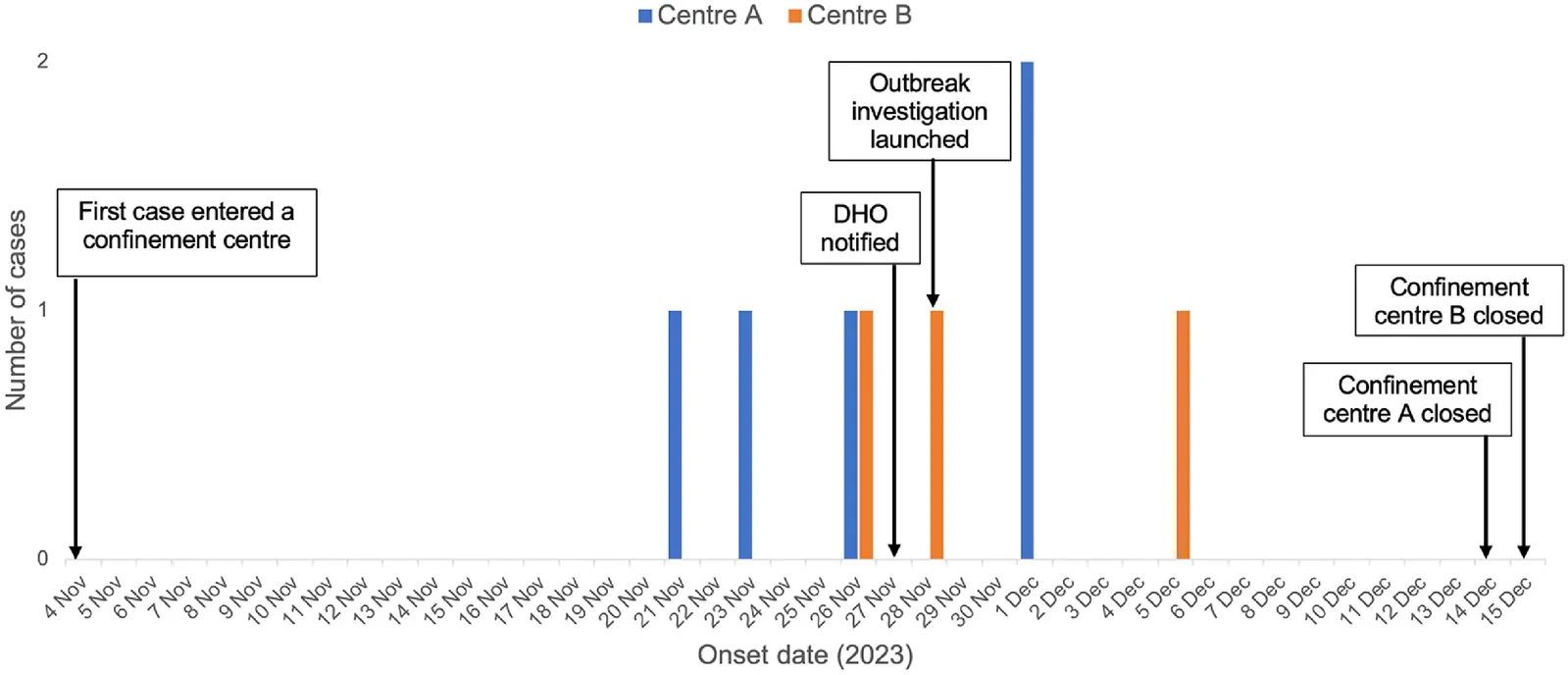
Epidemic curve of E11 outbreaks in two neonatal confinement centers (n = 10), Petaling District, Selangor, Malaysia; November to December 2023. Two of the 10 cases in this outbreak (one from each confinement center) were asymptomatic and had no onset date. Abbreviations: DHO, District Health Office; E11, echovirus 11.

The young age of the affected neonates highlights their vulnerability to enterovirus infection. Neonatal echovirus infections may be acquired during the peripartum period through maternal transmission or through nosocomial or community-associated exposure [22]. In this investigation, the temporal distribution of cases and the estimated incubation period were consistent with transmission within the confinement centers. The earliest identified cases in confinement centers A and B may have initiated subsequent transmission within their respective centers. Although cases occurred during the same period in both centers, no epidemiologic link was identified between the two clusters, suggesting that they may have resulted from separate introductions of E11.

The identification of asymptomatic neonates is particularly relevant to outbreak control because symptom-based surveillance alone may not identify all infected infants. Nevertheless, several neonates presented with central nervous system involvement, including meningoencephalitis, and E11 was detected in CSF specimens from affected neonates. The potential for unusual complications is further illustrated by a retrospective review of laboratory-confirmed E11 infections at Universiti Malaya Medical Centre in Kuala Lumpur between January 2023 and December 2024, which found that two of five hospitalized patients developed the rare complication of thrombotic microangiopathy [23]. These findings are consistent with the recognized potential of E11 to cause systemic and neurologic disease in neonates. The clinical presentation highlights the importance of considering enterovirus infection in neonates presenting with sepsis-like illness or neurologic manifestations, particularly when multiple cases occur in a common care setting.

The occurrence of E11 infection in a confinement center is of particular public health concern because these facilities provide care for multiple neonates in a shared environment during the early postnatal period. Neonates require frequent feeding, bathing, and diaper changes, resulting in repeated close contact with caregivers and shared equipment. Enteroviruses are primarily transmitted through the fecal–oral route and may also be spread through contaminated hands, fomites, and environmental surfaces. The frequent handling of diapers and other potentially contaminated materials may, therefore, provide opportunities for indirect transmission when appropriate hygiene measures are not consistently applied.

The environmental investigation identified several practices that could potentially facilitate transmission. These included close spacing between cots, consecutive handling of neonates by staff, the use of a shared bathing basin without immediate disinfection, delayed disposal of soiled diapers, and the absence of documented hand hygiene procedures between handling individual neonates. These observations do not establish a specific source or route of transmission, but they represent potential points for the interruption of fecal–oral and contact transmission. The similar findings observed in both centers are particularly noteworthy because they suggest that common operational practices in confinement centers may create comparable opportunities for transmission, even in the absence of a direct epidemiologic link between facilities.

The absence of E11 detection in staff hand swabs and the lack of reported fever, diarrhea, or medical leave among staff members does not completely exclude a role for staff in transmission. Enterovirus infections can be asymptomatic, and viral shedding may persist beyond the acute phase of illness [24]. Nevertheless, the available findings did not provide laboratory or epidemiologic evidence to implicate staff as a source of infection. This reinforces the importance of applying standard IPC precautions consistently to all caregivers, regardless of the presence or absence of symptoms.

The investigation also highlighted an important gap in the assessment of possible maternal transmission. Mothers were not sampled because they were asymptomatic and had limited contact with other neonates within the confinement centers. However, maternal sampling, particularly, of the mother of the earliest identified case, could have provided additional information regarding a possible source or direction of transmission. In addition, E11 infection in adults may occur after exposure to infected neonates [25]. Future investigations of neonatal E11 outbreaks should consider maternal specimens where they are clinically and epidemiologically appropriate, particularly, when investigating the earliest identified case.

The VP1 region is an established target for molecular identification and typing of enteroviruses because its sequence correlates well with enterovirus serotype. World Health Organization guidelines also identify VP1 as a useful target for molecular typing of human enteroviruses [26]. In this outbreak, molecular testing confirmed E11 infection, but phylogenetic analysis was not performed. Phylogenetic analysis could provide useful information on the genetic relatedness of E11 strains and help in understanding possible transmission pathways [27]. Future outbreak investigations should therefore consider phylogenetic analysis to support more detailed molecular epidemiologic investigations.

The absence of a national IPC protocol specifically tailored to confinement centers represents an opportunity to strengthen infection prevention measures in these settings. Standardized guidelines could address hand hygiene, environmental disinfection, diaper handling, feeding or bathing practices, the spatial arrangement of cots, staff training, and outbreak response procedures. Such guidelines could be adapted from established IPC principles used in neonatal health care settings while taking into account the operational characteristics of confinement centers. The response to this outbreak, including structured staff education, the revision of cleaning procedures, and subsequent reassessment of the centers, demonstrated the potential value of formalizing these practices.

The use of active case detection was also an important component of the outbreak response. Testing neonates in both confinement centers enabled the identification of asymptomatic infections that may otherwise have been missed through symptom-based surveillance. This approach is particularly relevant in neonatal settings, where clinical manifestations may be non-specific and overlap with other conditions, such as bacterial sepsis.

Several limitations should be considered. First, maternal specimens were not collected, limiting the assessment of possible maternal transmission. Second, environmental specimens were not collected before disinfection activities, preventing an assessment of environmental contamination with E11. Third, phylogenetic analysis was also not performed, so the genetic relatedness of E11 strains between the two centers could not be assessed.

Overall, the findings highlight the vulnerability of neonates to E11 infection and the potential for transmission in shared-care settings where multiple infants are cared for by the same staff. Although the two confinement centers were not epidemiologically linked, the presence of similar potential transmission risks in both facilities highlights the importance of standardized IPC practices in confinement centers. Early recognition, active case detection, appropriate laboratory investigation, effective environmental cleaning, and consistent hand hygiene are important components of outbreak control. Strengthening collaboration between hospitals, the DHO, laboratories, and confinement center operators, together with the development of standardized IPC guidelines for these facilities, may improve preparedness for future E11 outbreaks and other communicable disease threats.

## Conclusion

In conclusion, two concurrent E11 outbreaks occurred in confinement centers in Petaling District without an established epidemiological link. Similar IPC gaps were identified in both centers and might have facilitated viral transmission. The occurrence of asymptomatic and severe E11 infections highlights the importance of early case detection and appropriate investigation. Strengthening IPC practices, staff training, and collaboration between public health services, together with standardized IPC guidelines for confinement centers, may improve preparedness for future outbreaks.

## Funding

This research did not receive any specific grant from funding agencies in the public, commercial, or not-for-profit sectors.

## Ethical approval

This study was registered with the National Medical Research Registry and received ethical approval under NMRRID-24-00080-K1C.

## Declaration of generative AI and AI-assisted technologies in the writing process

The authors have nothing to disclose regarding the use of generative artificial intelligence or AI-assisted technologies in the preparation of this manuscript.

## CRediT authorship contribution statement

**Jun Fai Yap:** Data curation, Formal analysis, Methodology, Writing – original draft, Writing – review & editing. **Soo Cheng Lee:** Methodology, Project administration, Resources, Supervision, Visualization. **Zaharah Zainuddin:** Supervision, Validation, Writing – review & editing.

## Declaration of competing interest

The authors have no competing interests to declare.
